# Predictably unequal: understanding and addressing concerns that algorithmic clinical prediction may increase health disparities

**DOI:** 10.1038/s41746-020-0304-9

**Published:** 2020-07-30

**Authors:** Jessica K. Paulus, David M. Kent

**Affiliations:** Predictive Analytics and Comparative Effectiveness (PACE) Center, Institute for Clinical Research and Health Policy Studies (ICRHPS), Tufts Medical Center/Tufts University School of Medicine, Boston, MA USA

**Keywords:** Health care, Medical research

## Abstract

The machine learning community has become alert to the ways that predictive algorithms can inadvertently introduce unfairness in decision-making. Herein, we discuss how concepts of algorithmic fairness might apply in healthcare, where predictive algorithms are being increasingly used to support decision-making. Central to our discussion is the distinction between algorithmic fairness and algorithmic bias. Fairness concerns apply specifically when algorithms are used to support polar decisions (i.e., where one pole of prediction leads to decisions that are generally more desired than the other), such as when predictions are used to allocate scarce health care resources to a group of patients that could all benefit. We review different fairness criteria and demonstrate their mutual incompatibility. Even when models are used to balance benefits-harms to make optimal decisions for individuals (i.e., for non-polar decisions)–and fairness concerns are not germane–model, data or sampling issues can lead to biased predictions that support decisions that are differentially harmful/beneficial across groups. We review these potential sources of bias, and also discuss ways to diagnose and remedy algorithmic bias. We note that remedies for algorithmic fairness may be more problematic, since we lack agreed upon definitions of fairness. Finally, we propose a provisional framework for the evaluation of clinical prediction models offered for further elaboration and refinement. Given the proliferation of prediction models used to guide clinical decisions, developing consensus for how these concerns can be addressed should be prioritized.

## Background

“…you do not really understand a topic until you can teach it to a mechanical robot”—Judea Pearl and Dana Mackenzie^1^.

Consistent and substantial differences in the treatment of medical conditions in patients who differ by race/ethnicity or by sex have raised concern that clinician bias may contribute to disparities in healthcare^2–4^. The emergence of artificial intelligence holds promise that computer-based algorithms may ameliorate human biases and possibly attenuate health disparities^5^. However, computer scientists have recently become alert to the possibility that predictive algorithms can inadvertently introduce unfairness in decision-making. This is a major concern as algorithmic technologies have permeated many important sectors: criminal justice (e.g., predicting recidivism for parole decisions); the financial industry (e.g., credit worthiness); homeland security (e.g., “no fly” lists); and targeted ads (e.g., job listings). Indeed, legislation has recently been proposed in the U.S. that would direct the Federal Trade Commission to require the assessment of algorithmic fairness and bias by entities that use, store, or share personal information for algorithmically supported decision-making^6^.

Despite the broader awareness of the importance of algorithmic fairness, and the rapidly expanding impact of algorithmic prediction in healthcare, how principles of algorithmic fairness might apply in clinical decision-making has received little attention in the medical literature^7,8^. In this perspective, we review methodological research from the computer science literature and relevant epidemiological principles, to clarify when fairness concerns might be germane and to introduce a practical framework for evaluating algorithmic bias and fairness in clinical decision-making and prediction in healthcare. While we focus on race, the discussion may extend to other classes (such as ethnicity, religion or creed, sex, national origin, etc.) legally protected against discrimination in certain settings. This perspective is intended for those stakeholders who are developing algorithms (e.g., clinical researchers, medical informaticians), as well as users of models, such as healthcare administrators, clinicians, and payers.

## The fundamental problem of prediction and prejudice: reference class forecasting is discrimination by group membership

Machine learning and statistical algorithms make predictions on individuals using mathematical models that are not explicitly programmed, but rather are developed using statistical rules that associate variables (or features) with outcomes (or labels) within a training data set. Machine learning is thus a form of “reference class forecasting”^9^ whereby an individual’s risk of a given outcome is estimated by examining outcome rates in a group of others with “similar” features. Because people have many different attributes, and because there are many different approaches to modeling, there are many different ways to define similarity; thus, any given individual’s “risk” is model-dependent. Each different way of defining similarity leads to a different risk estimate–and often a very different risk estimate–for a given individual^10,11^.

The fact that “risk” is not a property that can be objectively measured in an individual (like blood pressure or cholesterol)—but instead can only be estimated in a group of other individuals judged to be similar in a set of selected features—suggests the overlap between the concepts of reference class forecasting and prejudice: in both, an individual’s disposition is determined by that person’s group membership.

A key statistical measure of model performance is how well the model discriminates between those who have the outcome and those who do not. Disentangling the two meanings of “discrimination”—discernment between individuals’ risk of a future event on the one hand and unfair prejudice leading to inequity on the other (akin to what economist Thomas Sowell has referred to as Discrimination I and Discrimination II, respectively^12^)—is central to understanding algorithmic fairness, and more deeply problematic than generally appreciated.

## Common sense fairness criteria are superficially appealing but mutually conflicting

The specter of “machine bias” was highlighted in 2016. Using data from over 7000 arrests, an investigative report showed that a commercial software (COMPAS) used to predict the risk of criminal re-offense assigned a higher risk of reoffending to black defendants than to whites, leading to potentially longer sentences. This was true even among those who did not subsequently recidivate, i.e., whose “true” risk is (retrospectively) 0%. These disparities emerged even though the algorithm was “race-unaware”—i.e., race was not explicitly coded for in the statistical model (as it is potentially illegal to use protected characteristics in sentencing decisions); other features correlated with race were included. The observed unequal error rates between blacks and whites—even among those whose future behavior was the same—corresponds to common sense notions of unfairness. It has been argued that unequal error rates also align with legal definitions of discrimination through “disparate impact”^13^, which proscribes practices that adversely affect one group of people more than another, even when the rules (or the statistical models) are formally neutral across groups^14^. Nonetheless it’s important to bear in mind that fairness and the legal standard of disparate impact are not purely statistical concepts, and involve ethical, political and constitutional concerns^15^.

However, the software developers argued that the model is fair as it had similarly good calibration across both white and black populations. Calibration refers to the agreement between observed outcomes and predictions. For example, if we predict a 20% risk of recidivism in a group of subjects, the observed frequency of recidivism should be ~20 out of 100 individuals with such a prediction. Like unequal error rates, calibration also appears to conform to informal notions of fairness in that a given score from a prediction model should correspond to the same probability of the outcome, regardless of group membership (known as the test fairness criteria).

**Fig. 1:**
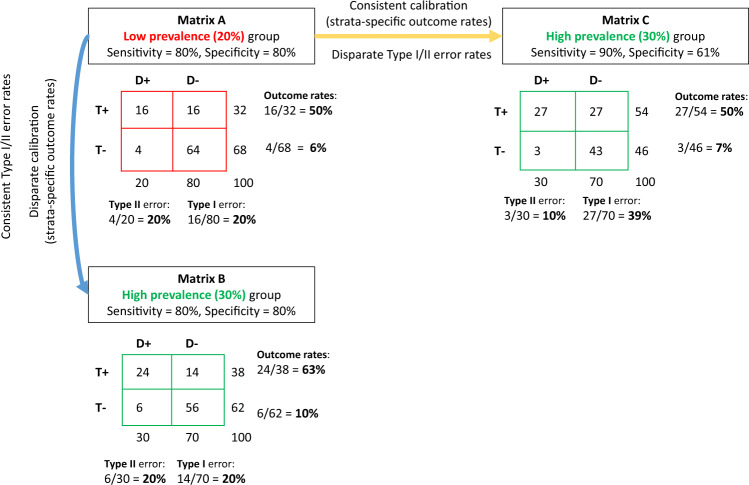
Mutual incompatibility of fairness criteria. For two groups with different outcome rates, a predictive test can have consistent error rates or consistent calibration but not both. We present outcomes using coarsened prediction scores, thresholded to divide the population (*N* = 100) into low and high risk strata. Confusion matrices for a low prevalence group with a 20% outcome rate (Matrix A, red) and a high prevalence group with a 30% outcome rate (Matrices B and C, green) are shown. For the low prevalence group, a predictive test with an 80% sensitivity and specificity identifies a high risk (test+) strata with an outcome rate of 50% (i.e., the positive predictive value) and a low risk (test−) strata with an outcome rate of ~6% (i.e., the false omission rate). However, as shown in Matrix B, the same sensitivity and specificity in the higher prevalence group gives rise to outcome rates of ~63% and ~10% in the high and low risk-strata, respectively. This violates the criterion of test fairness, since the meaning of a positive or negative test differs across the two groups. Holding risk-strata specific outcome rates constant would require a higher sensitivity and lower specificity (Matrix C). This violates the fairness criteria of equalized error rates. For example, the Type I error rate (i.e., the false positive rate) would almost double from 20% in the low prevalence population to ~39% in the higher prevalence population. The diagnostic odds ratio was fixed at ~16 across this example, whole numbers are used to ease interpretation.

Subsequently, it was demonstrated mathematically that these two fairness criteria–equalized error types and test fairness–cannot both be satisfied when the outcome rates differ across the two groups (except in the unrealistic circumstance of perfect prediction), leading to the conclusion that unfairness is inevitable^13,16^. Figure 1 provides a numerical illustration showing that, when outcome rates vary across two groups, a predictive test can have consistent error rates or consistent calibration across groups but not both. Because there are many different fairness criteria (Table 1), and these may be mutually incompatible^17–19^, prioritizing across criteria necessarily involves a value judgment and may be sensitive to various contextual factors.

**Table 1:** Candidate criteria to assess algorithmic fairness.

Criterion	Explanation	
Unconditional equality of classification or predicted probabilities		
Statistical parity also known as: demographic parity or disparate impact	Participants/patients have equal probability of being assigned to the positive predicted class, or the same average predicted probability, for all values of the protected attribute. A violation of statistical parity is probably the most common (and least rigorous) notion of unfairness. Indeed, satisfying statistical parity often requires positive discrimination, i.e., disparate treatment for different values of the protected attribute. A variant of this criterion (conditional statistical parity) requires equal probability of being assigned to the positive predicted class conditional on other allowable variables. Complex fairness concerns are at issue in determining allowable versus unallowable factors for conditioning. When one conditions on all causal variables, this criteria converges with disparate treatment (see below).	
Equality of classification/predictions conditioned on observed outcome (see blue arrow in Fig. 1)		
Classification	Equalized odds also known as: error rate balance	The probability of being correctly classified conditional on the outcome should be the same for all values of the protected attribute.
Predicted probability	Balance on the positive class	The algorithm produces the same average prediction (or score) for participants/patients with the outcome across all values of the protected attribute. For a binary prediction (i.e., a classifier), this is equivalent to maintaining equal sensitivity and type II error (false negative rates).
	Balance on the negative class	The algorithm produces the same average prediction (or score) for participants/patients without the outcome across all values of the protected attribute. For a binary prediction (i.e., a classifier), this is equivalent to maintaining equal specificity and type I error (false positive rates).
Equality of outcomes conditioned on classification/prediction (see orange arrow in Fig. 1)		
Classification	Positive predicted value (PPV)	For participants/patients assigned to the positive class, observed outcome rates (e.g., PPV) are the same across values of the protected attribute.
	Negative predicted value (NPV)	For participants/patients assigned to the negative class, observed outcome rates (e.g., 1-NPV, or the false omission rate) are the same across values of the protected attribute.
Predicted probability	Calibration also known as: test fairness	An algorithm is said to have good calibration if, for any given subgroup with a predicted probability of X%, the observed outcome rate is X% for all values of the protected attribute. For any single threshold, a well-calibrated prediction model will never have the same sensitivity and specificity for two groups with different outcome rates.
Causal definitions of fairness		
Disparate Treatment	A causal notion of fairness; otherwise similar individuals should not be treated differently due to having different protected attributes. Causal notions of unfairness are the most rigorous and least controversial, but are unidentifiable in observational data.	

The impossibility of simultaneously satisfying the various fairness criteria points both to the inevitability of unfairness (defined by heterogeneous “common sense” outcomes-based measures) and to the limited validity, authority and usefulness of these measures. If we start from the premise that fair and unbiased decision-making is possible in theory, the impossibility results suggest that unequal outcomes will emerge from both fair and unfair decision-making. To satisfy more stringent, narrow, and rigorous definition of unfairness, it is not enough to observe differences in outcomes – one must understand the causes for these outcome differences. Such a causal concept of fairness is closely aligned to the legal concept of disparate treatment (Table 1)^20^. According to causal definitions of fairness, similar individuals should not be treated differently due to having certain protected attributes that qualify for special protection from discrimination, such as a certain race/ethnicity or gender. However, causality is fundamentally unidentifiable in observational data, except with unverifiable assumptions^20,21^. Thus, we are more typically stuck with deeply imperfect but ascertainable criteria serving as (often poor) proxies for causal fairness.

## A fundamental conflict in fairness principles

The conflict between fairness criteria reflects the fact that criteria based on outcomes do not correspond to causal notions of fairness. While a complete understanding of the true causal model determining an outcome (or label) promises in theory to provide the bedrock to determine fair processes for prediction and decision-making (by permitting the disentangling of legitimate causal attributes from illegitimate race-proxies), we note that differing conceptions of fairness would still ensure that fairness definitions remain deeply contested. There are two competing principles or goals in antidiscrimination law^15^: anticlassification and antisubordination. The goal of anticlassification is to eliminate the unfairness individuals experience due to bias in decision-makers’ choices, whereas antisubordination seeks to eliminate status-based inequality across protected classes. Enforcing balance in outcomes or results can only indirectly address anticlassification concerns (if at all)—since large differences in group outcomes can arise with or without biased decision-making. Conversely, ensuring fair processes is unlikely to satisfy those who adhere to the antisubordination principle, since this requires adjudicating the degree of difference between groups that a fair society should tolerate.

## Fairness concerns are not clearly relevant for all decisional contexts

In addition to the limited validity and authority of proposed results-focused fairness criteria, it is important to recognize the limits of their relevance across decision contexts. In particular, the contexts described above (such as in the criminal justice system) differ from that which often defines decisions for medical decision-making. Fairness concerns are important when decisions must arbitrate between competing interests among different parties, in ways that they do not for other types of decisions. According to the Stanford Encyclopedia of Philosophy:

“Issues of justice arise in circumstances in which people can advance claims… that are potentially conflicting, and we appeal to justice to resolve such conflicts by determining what each person is properly entitled to have. In contrast, where people’s interests converge, and the decision to be taken is about the best way to pursue some common purpose… justice gives way to other values”^22^.

**Fig. 2:**
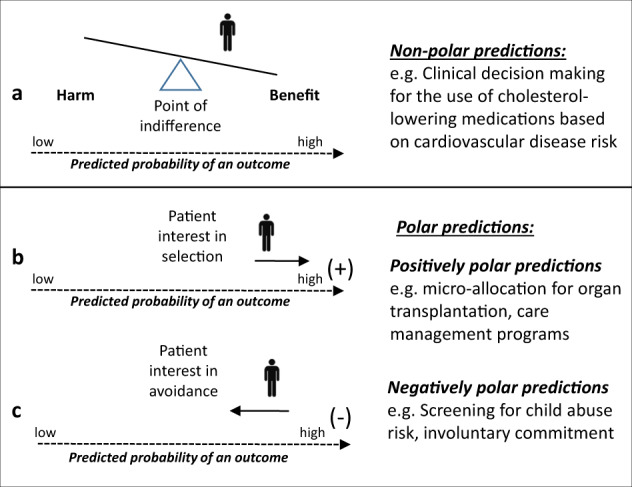
Non-polar and polar prediction-supported health care decisions. Understanding the specific decisional context of a prediction-supported decision in healthcare is necessary to anticipate potential unfairness. In the medical context—particularly in the shared decision-making context—patients and providers often share a common goal of accurate prognostication in order to help balance benefits and harms for care individualization. Predictions supporting decisions in this context may be described as “non-polar” (**a**). On the other hand, when one “pole” of the prediction is associated with a clear benefit or a clear harm, predictions may be described as “polar” in nature. In cases of polar predictions, the decision maker’s interest in efficient decision making (i.e. based on accurate prognostication using all available information) is not aligned with the subject’s interest to have either a lower (e.g. screening for abuse risk) or higher (e.g. microallocation of organs) prediction. “Positively” polar predictions correspond to those where patients may have an interest to be ranked high to receive a service that may be available only to some of those who can potentially benefit (**b**). This is in distinction to “negatively” polar predictions, in which prediction is used for the targeting of an intervention perceived as punitive or coercive (e.g. such as involuntary commitment, screening for child abuse or mandatory quarantining those at high infectious risk) (**c**). Issues of fairness pertain specifically to predictions used in decisional contexts that induce predictive polarity—since these are contexts in which people advance claims that are potentially conflicting.

In many of the non-medical examples, there are clearly competing interests—for example, between society’s need for safety and security and an individual’s claim to freedom and freedom from harassment; between a lending institution’s responsibility to remain financially healthy and an individual’s desire for a loan. In these conditions, predictions can be said to be “polar”—i.e., one end of the probability prediction is linked to a decision that is (from the perspective of the subject) always favorable or unfavorable^23^. It is always better to get a lower recidivism score or a higher credit rating, for example, from the perspective of the individual whose score or rating is being predicted. In this context, the decision-maker’s interest in efficient decision-making (i.e., based on accurate prognostication using all available information) is not aligned with the subject’s interest in receiving the lowest (or highest) possible risk prediction. However, in the medical context accurate prognostication helps decision-makers appropriately balance benefits and harms for care individualization—the common goal of the patient and provider. When the clinician/decision-maker’s and patient’s interests are aligned (or when the patient is in fact the decision-maker), and when race has important predictive effects not captured by other variables included in the model, including race/ethnicity as a variable in models used for this purpose improves predictions and decisions for all groups. Prediction supporting decisions in this context may be described as “non-polar” (Fig. 2a).

But in medicine too, there are contexts where the interests of the clinician/decision-maker and the patient diverge, such as when predictions are used to prioritize patients for rationed services that might benefit a broader population (e.g. organ transplantation, disease management programs, or ICU services). We label predictions used for microallocation of scarce medical resources as “positively” polar, indicating that patients may have an interest to be ranked high to receive a service that may be available only to some of those who can potentially benefit (Fig. 2b). This is in distinction to “negatively” polar predictions, in which prediction is used for the targeting of an intervention perceived as punitive or coercive (e.g. such as involuntary commitment, screening for child abuse, or quarantining patients at high infectious risk; Fig. 2c). Use of algorithms for microallocation (i.e. rationing based on individual characteristics) is likely to play a larger role for population health management in accountable care organizations or value-based insurance design. Allocating scarce health care resources on the basis of a protected characteristic—or using such characteristics as the basis for other “polar” decisions–appears to have similar fairness concerns as many of the high profile non-medical examples.

## Learning from biased data

While fairness concerns are alleviated in the setting of non-polar prediction, additional problems arise when the data itself are biased or mislabeled across classes (for polar and non-polar prediction alike). We use the term algorithmic bias (in distinction to fairness) specifically to refer to these issues related to model design, data and sampling that may disproportionately affect model performance in a certain subgroup. Consider, for example, prediction models developed on routinely collected electronic health data to target cancer screening of populations with higher cancer rates. Because cancer diagnosis is an imperfect proxy for cancer incidence, rates of “surveillance-sensitive” cancers (e.g., thyroid and breast cancer) are inflated in affluent compared to underserved communities^24^. This could lead to the mis-targeting of screening to the over-served, thereby establishing a continuously self-reinforcing positive feedback loop.

Similarly, consider mortality predictions that might support decisions in the intensive care unit, such as the determination of medical futility. Using “big data” across multiple health systems with different practice patterns might lead to the assignment of higher mortality probabilities to the types of patients seen at institutions with less aggressive approaches or lower quality care. Collinearity between patient factors and care factors can bias prognostication and lead again to a self-reinforcing loop supporting earlier withdrawal of care in the underserved. Observed mortality is an imperfect proxy for mortality under ideal care, the true outcome of interest when constructing models for futility.

The above are examples of label bias, which arises when the outcome variable is differentially ascertained or otherwise has a different meaning across groups. There may also be group differences in the meaning of predictor variables; this is known as feature bias. For example, feature bias may be a problem if diagnoses are differentially ascertained or thresholds for admission or healthcare-seeking differ across groups in the training data and model features (prediction variables) include prior diagnosis or previous hospitalization. Label and feature biases, as well as differential missingness, can contribute to violations of subgroup validity, which arise when models are not valid in a particular subgroup. Subgroup validity may also be a concern in the context of sampling bias, where a minority group may be insufficiently represented in model development data (e.g., certain ethnic groups in the Framingham population^25^) and the model might be tailored to the majority group. When effects found in the majority group generalize well to the minority group, this is not problematic but generalization across groups should not be assumed. Sampling bias was a well-known issue with the highly influential Framingham Heart Study, which drew its study population from the racially homogeneous town of Framingham, Massachusetts—and consequently can lead to both over- and under-treatment of certain ethnic minorities^25,26^. More recently, the emergence of polygenic risk scores derived largely on European populations have been shown to generally perform very poorly on non-European populations^27^. For similar reasons, there are concerns about the representativeness of the Precision Medicine Initiative (the “All of Us” Study^28^).

## Should the use of protected characteristics in clinical prediction models (CPM) differ for polar versus non-polar predictions?

Currently, there is no consensus or guidance on how protected characteristics–race in particular– should be incorporated in clinical prediction^29^. Previous work found race to be included only rarely in cardiovascular disease prediction models, even when it is known to be predictive^30^. Several authors explicitly acknowledged excluding race from prediction models due to concerns about the implications of “race-based” clinical decision-making^31^.

We have previously argued that much of the reluctance to use race in prediction models stems from overgeneralization of its potentially objectionable use in polar predictions in non-medical settings to its use for non-polar predictions in medical settings^29^. The ethical issues involved in using race or race proxies to move a person up or down a prediction scale with a clear directional valence (liberate versus incarcerate; qualify versus reject a loan application; receive versus not receive an available donor organ) are clearly different than for optimizing one’s own decisions about whether to take or not take a statin; whether percutaneous coronary intervention might be better than coronary artery bypass; whether medical therapy might be superior for carotid endarterectomy and so forth.

For these latter non-polar decisions, a mature literature exists for how to evaluate prediction models to optimize decision-making in individual patients^32^. When race is importantly predictive of health outcomes (as it often is), excluding race from a model will lead to less accurate predictions and worse decision-making for all groups. In particular, “race-unaware” models (i.e., models that exclude race) will often especially disadvantage those in minority groups, since predictions will more closely reflect outcomes and associations for patients in the majority. Indeed, race is used explicitly in popular prediction models that inform the need for osteoporotic^33^, breast^34–37^ and prostate cancer screening^38,39^; statin use for coronary heart disease prevention^40,41^ and other common decisions.

For polar predictions, however, there are efficiency-fairness trade-offs that are not germane in the non-polar context. To take a non-medical example, developing a model which predicted loan default, use of variables such as “income,” “assets,” and “credit history” might be uncontroversial—even if race-correlated. However, even if using race (or race proxies without a clear causal link to the outcome) in addition to these variables substantially improved model performance and increased the efficiency of decision-making and the overall net economic benefits, the use would still be unethical and violate the disparate treatment criteria. Similar principles presumably apply regarding the use of protected characteristics when using predictions to ration resources decisions in health care.

## Putting it all together: towards a framework for bias and for unfairness

The above discussion suggests different considerations and approaches for polar and non-polar predictions. In the former context, we argue, both bias and fairness concerns apply whereas ensuring an unbiased model is sufficient in the latter.

### How to ensure unbiased models

**Fig. 3:**
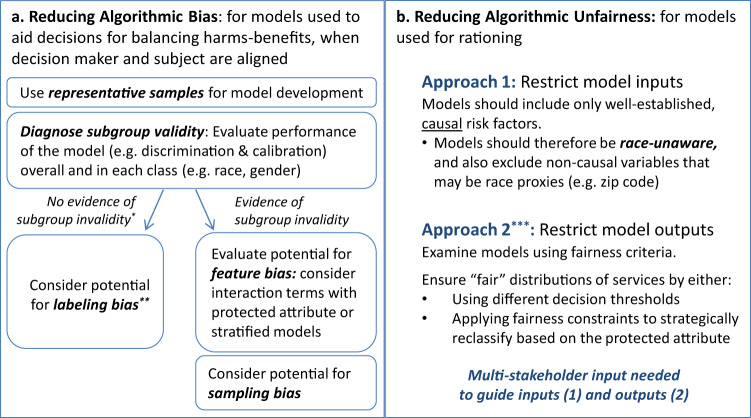
Mitigating algorithmic bias and unfairness in clinical decision-making. Bias arises through differential model performance across protected classes, such as across racial groups. **a** It is a concern in both polar and non-polar decision contexts and can be addressed by “debiasing” predictions, typically through the explicit encoding of the protected attribute to ameliorate subgroup validity issues, or by the more thoughtful selection of labels (in the case of labeling bias). Fairness concerns are exclusively a concern in polar decision contexts, and may persist even when prediction is not biased. **b** There are two broad and fundamentally very different unfairness mitigation approaches: (1) an input-focused approach, and (2) an output-focused approach (Fig. 3b). The goal of the input-focused approach is to promote class-blind allocation by meticulously avoiding the inclusion of race or race proxies. The output-focused approach evaluates fairness using criteria such as those described in Table 1 and Fig. 1. Fairness violations can be (partially) addressed through the use of “fairness constraints” (which systematically reclassify participants/patients to equalize allocation between groups) or by applying different decision thresholds across groups.

With the exception of label bias, which can be difficult to diagnose with the data because the outcome itself has a different meaning across groups (and thus recognition of label bias requires external knowledge about how the data are ascertained), the above subgroup validity issues can generally be diagnosed by examining model performance separately in each of the groups (Fig. 3a). When a model is found to be poorly calibrated in a subgroup, provided the minority populations are sufficiently represented in the data, this can often be addressed by the inclusion of a main effect for group status; inclusion of selected interactions between group status and other features; or developing stratified models. Indeed, the widely-used Pooled Cohort Equation for coronary heart disease prediction addressed the subgroup validity issues identified in the Framingham score (i.e., poor model performance in ethnic minorities) by developing separate models for whites and African-Americans^42^.

Labeling bias should be anticipated whenever a proxy is used as the outcome or label. Problems with proxy labels are very similar to the well-described, familiar problem with surrogate outcomes in clinical research^43,44^. Like surrogate outcomes, proxy labels can often seem compellingly, persuasively similar to the outcome of interest and nevertheless be very misleading. The remedy here is to try to pick a better label (i.e., outcome definition). A high profile example of this was recently reported in which an algorithm used to target services to those with high health needs used future health care costs as a proxy for need. The bias was detected because black patients were sicker than similarly scored white patients, and the algorithm was remedied through the use of a better label that more directly captures health need^45^.

### Addressing fairness concerns

Reducing model bias and differential performance may be insufficient to eliminate fairness concerns in decision contexts characterized by predictive polarity (such as when predictions are used to ration health care resources), where unambiguously favorable (or unfavorable) decisions are associated with a higher (or lower) score. Here, we identify two broad and fundamentally very different unfairness mitigation approaches: (1) an input-focused approach, and (2) an output-focused approach (Fig. 3b).

The input-focused approach relies on model transparency; it loosely aligns with anticlassification goals and avoidance of disparate treatment since it promotes class-blind allocation by meticulously avoiding the inclusion of race or race proxies. Since any variable can be correlated with race and therefore serve as a proxy, only highly justified, well-established causal variables should be included in the model. The use of “high dimensional” or “black box” prediction techniques typically favored in the machine learning community are generally problematic (since these approaches can predict race through other variables, whether or not race is explicitly encoded)—although methods that have been proposed to make these models more transparent have recently been adapted to address fairness^46^.

In contrast, the output-focused approach does not restrict model development, but relies on an evaluation of model predictions using outcomes-based fairness criteria (Table 1) and seeks to mitigate fairness concerns by making use of “fairness constraints”. These constraints can be understood as formalized “affirmative action” rules to systematically reclassify subjects in an attempt to equalize allocation between groups^19,47^. This approach aligns loosely with the legal concepts of antisubordination and disparate impact; it has the disadvantage that there is no agreed upon mathematical solution to define fairness. Because value judgments are key for any approach to fairness, robust input from a diverse set of stakeholders who are developing, using, regulating and are affected by clinical algorithms should be sought. The stakeholders include patients and their advocates, model developers (e.g., clinical researchers, informaticians), model users/deployers (e.g., healthcare administrators, clinicians, payers), and health policy, ethical and legal experts. Application of results-oriented criteria requires standards or consensus regarding what degree of disparity in allocation of health care resources across groups might be intolerable.

## Limitations

To be sure, the framework we introduce is simplified and provisional, and is intended as a starting point. Adding further complexity is that some predictive algorithms are applied in different decisional contexts with different ethical concerns. For example, the estimated GFR equations, (which are race-aware) may be used to inform both resource prioritization (e.g., transplant lists) and for appropriate medication dosing^48^. Sometimes the polarity of a prediction may be non-obvious. We also acknowledge that some objections to the use of race as a variable in prediction models have little to do with unfairness as described here^49^. Finally, we wish to underscore the political and legal complexities of identifying and mitigating algorithmic disparities and the need to integrate statistical and legal thinking –amongst other stakeholders - in devising remedies.

## Conclusion

People are often told–either by elders or by experience itself–that life is unfair; now there is mathematical support^16^ for that gloomy bit of wisdom. Yet fairness is a central preoccupation of any decent society. While there is no universally accepted algorithmic solution to the problem of unfairness, the problem also cannot be solved by replacing algorithms with a human decision-maker—just obscured. Formalizing predictions opens the issues up to communal (and mathematical) scrutiny, permitting us, for example, to more precisely understand the conflict between competing fairness notions and the limitations of these notions. This is an essential, though insufficient, step in developing consensus about how to impose human values on agnostic, data-driven algorithms, and how to supervise these algorithms to ensure fairer prediction and decision-making in healthcare and elsewhere. More rigorous and narrow (e.g., causal) definitions of unfairness might be a part of the answer, though a wholly technical solution seems unlikely. A set of principles^50^ has been articulated to provide guidance to those developing and disseminating algorithms (Box 1)—principles that may ultimately get encoded into law^6^. If we can figure out how to encode fairness into computer programs, we may yet come to a deeper understanding of fairness, algorithmic and otherwise.

Box 1. Principles for accountable algorithms developed by FAT/ML (adapted from fairness, accountability, and transparency in machine learning)^50^

**Table:** 

Responsibility	Identify a person/persons and process for monitoring and remedying issues related to the algorithm
Explainability	Ensure that the algorithm is understandable to users and stakeholders
Accuracy	Consider sources and impact of possible errors
Auditability	Establish a system that will allow transparent public auditing of the algorithm
Fairness	Anticipate and assess the potential for algorithmic unfairness
